# *Helicobacter pylori *genome variability in a framework of familial transmission

**DOI:** 10.1186/1471-2180-7-54

**Published:** 2007-06-11

**Authors:** Mårten Kivi, Sandra Rodin, Ilya Kupershmidt, Annelie Lundin, Ylva Tindberg, Marta Granström, Lars Engstrand

**Affiliations:** 1 Department of Microbiology, Tumor and Cell Biology (MTC), Karolinska Institutet, 171 77 Stockholm, Sweden; 2 Department of Bacteriology, Swedish Institute for Infectious Disease Control (SMI), 171 82 Solna, Sweden; 3 Department of Biotechnology, Royal Institute of Technology (KTH), 100 44 Stockholm, Sweden; 4 Agilent Technologies, 395 Page Mill Rd., Palo Alto, CA 4306, USA; 5 Department of Medical Epidemiology and Biostatistics (MEB), Karolinska Institutet, 171 77 Stockholm, Sweden

## Abstract

**Background:**

*Helicobacter pylori *infection is exceptionally prevalent and is considered to be acquired primarily early in life through person-to-person transmission within the family. *H. pylori *is a genetically diverse bacterial species, which may facilitate adaptation to new hosts and persistence for decades. The present study aimed to explore the genetic diversity of clonal isolates from a mother and her three children in order to shed light on *H. pylori *transmission and host adaptation.

**Results:**

Two different *H. pylori *strains and strain variants were identified in the family members by PCR-based molecular typing and sequencing of five loci. Genome diversity was further assessed for 15 isolates by comparative microarray hybridizations. The microarray consisted of 1,745 oligonucleotides representing the genes of two previously sequenced *H. pylori *strains. The microarray analysis detected a limited mean number (± standard error) of divergent genes between clonal isolates from the same and different individuals (1 ± 0.4, 0.1%, and 3 ± 0.3, 0.2%, respectively). There was considerable variability between the two different strains in the family members (147 ± 4, 8%) and for all isolates relative to the two sequenced reference strains (314 ± 16, 18%). The diversity between different strains was associated with gene functional classes related to DNA metabolism and the cell envelope.

**Conclusion:**

The present data from clonal *H. pylori *isolates of family members do not support that transmission and host adaptation are associated with substantial sequence diversity in the bacterial genome. However, important phenotypic modifications may be determined by additional genetic mechanisms, such as phase-variation. Our findings can aid further exploration of *H. pylori *genetic diversity and adaptation.

## Background

The bacterium *Helicobacter pylori *infects the gastric mucosa of about half of the world's population, rendering it one of the most common bacterial infections in humans [1]. The infection is associated with low socioeconomic status and is usually acquired in early childhood [1-3]. In the absence of consistent and verified environmental reservoirs, the transmission is considered to occur primarily from person-to-person. Infected family members, especially mothers and siblings, are strong risk factors for children to be infected [3,4]. Furthermore, clonal bacterial isolates have essentially only been identified in family members or the same person [5-8]. Accordingly, intrafamilial transmission has been postulated as the predominant mode of *H. pylori *dissemination.

A hallmark of *H. pylori *is its ability to maintain an infection throughout the lifetime of an individual. The infection is accompanied by gastritis and, following persistent infection, a subset of infected individuals develops peptic ulcer or gastric cancer [1]. Another distinguishing feature of the bacterium is its extensive genetic diversity, originating in acquisition, deletion, rearrangement and point mutation of DNA sequences [6,9-16]. The diversity may facilitate adaptation to new hosts and persistence for decades despite a changing gastric milieu, consequently contributing to the high prevalence of the infection worldwide. Thus, understanding the characteristics and mechanisms of *H. pylori *variability in a framework of transmission and host adaptation is a central and basic issue.

Previous studies have described considerable whole-genome variability between unrelated isolates from different individuals [9,11,13-16] and significantly less diversity of clonal isolates within the same individual [9,16-18]. The present study aimed, for the first time, to explore the genetic diversity of clonal isolates within and between members of a family by sequencing and comparative genomic microarray hybridizations in order to shed light on *H. pylori *transmission and host adaptation.

## Results

*H. pylori *isolates from the corpus sample of the mother (biopsy 16) and the antrum samples of her three children (biopsies 13, 125, 24a) yielded similar patterns by RAPD molecular typing (RAPD type B) (Table 1). The mother also harbored an additional strain in the antrum sample (biopsy 15, RAPD type A). At a later stage, a previously typed antrum sample from another biopsy of the mother was retrieved [5], whereby a mixture of clones of RAPD type A and B was identified.

**Table 1:** Subjects and samples in the present study

Family member	Age	Birth area	Biopsy:isolates (location)	RAPD^1 ^type
Mother	39	South America	15:1–11 (antrum)	A^2^
			16:1–13 (corpus)	B
Child A	21	South America	13:1–11 (antrum)	B
Child B	19	South America	125:1–12 (antrum)	B
Child C	13	Sweden	24a:1–12 (antrum)	B

^1^Random amplified polymorphic DNA molecular typing

^2^Another antrum biopsy of the mother contained isolates of both RAPD type A and B.

The clustering of the isolates was confirmed and clonal variants were identified by sequencing of five loci in three isolates per biopsy (Table 2) (Additional file 1). The RAPD type A isolates typically contained the same alleles, which differed from those of the RAPD type B isolates. The *hsdS5 *sequences of type A isolates exhibited large differences compared to type B isolates and were aligned separately. The *cag2 *(HP0521) primers did not yield any product for the type B isolates, but instead the HP0521B gene was detected. The HP0521B gene has been identified as a new gene at the site of *cag2 *(HP0521) and is present in approximately half of Swedish clinical isolates [19]. Furthermore, the *flaB *and *mutY *products exhibited sequence variation between type A and type B isolates, but for the latter locus some of the divergence was shared between the type A isolates and the type B isolates of one child. All isolates yielded the same *ureI *sequences except for a few nucleotide differences in two of the type B isolates of the mother.

**Table 2:** Sequence differences in base pairs (bp) by isolate and amplified fragment

Isolate	Sequence differences by PCR fragment (length of sequence in bp)				
	
(RAPD type)	*hsdS *(571/607)^1^	*cag2 *(330/475)^2^	*flab *(487)	*mutY *(439)	*ureI *(576)
15:7 (A)	0^1^	0^2^	27^3^	19^4^	0
15:8 (A)	0^1^	0^2^	27^3^	19^4^	0
15:10 (A)	0^1^	0^2^	27^3^	19^4^	0
16:1 (B)	0	0	0	0	6^5^
16:3 (B)	0	0	0	0	0
16:13 (B)	0	0	0	0	1^5^
13:5 (B)	0	0	0	13^4^	0
13:10 (B)	0	0	0	13^4^	0
13:11 (B)	0	0	0	13^4^	0
125:3 (B)	0	0	0	0	0
125:7 (B)	0	0	0	0	0
125:11 (B)	0	0	0	0	0
24a:5 (B)	0	0	0	0	0
24a:10 (B)	0	0	0	0	0
24a:12 (B)	0	0	0	0	0

Sequence differences were assessed between 15 *H. pylori *isolates, three isolates from each of biopsy 15 and 16 from the mother and of biopsy 13, 125 and 24a from the three children.

^1^The 571 bp fragments from isolates from biopsy 15 differed significantly from the 607 bp fragments from the other isolates and were aligned separately.

^2^The 330 bp *cag2 *(HP0521) fragments were obtained from isolates from biopsy 15, while 475 bp fragments of the HP0521B gene were amplified from the other isolates, and separate alignments were performed.

^3^Sequences are identical within biopsy 15.

^4^Sequences are identical within biopsy 15 and 13, respectively, and the difference of 13 bp is shared between isolates from biopsy 15 and 13.

^5^The difference of 1 bp is not shared between isolates 16:1 and 16:13

The microarray data grouped the isolates in the same fashion as RAPD and sequencing. The diversity as measured by microarray was assessed quantitatively for all 105 individual combinations of test isolates and relative to the reference strains (Table 3) (Additional file 2). Of the 1,745 genes analyzed, a mean number of 1 ± 0.4 (0.1%) genes were classified as divergent in the 15 comparisons between the three isolates from the same biopsy. The diversity between type B isolates from different family members was estimated to a mean of 3 ± 0.3 (0.2%) divergent genes in the 54 corresponding comparisons. In the 36 comparisons between isolates of type A (biopsy 15) and type B (biopsies 16, 13, 125 and 24a), a mean of 147 ± 4 (8%) genes were classified as divergent. The mean number of divergent genes for each of the 15 isolates relative to the reference strains was 314 ± 16 (18%).

**Table 3:** Number of microarray divergent genes averaged by biopsy

Biopsy (RAPD	Mean number of divergent genes of 1,745 (± SE^1^)					
	
type of isolates)	15 (A)	16 (B)	13 (B)	125 (B)	24a (B)	Reference 26695/J99
15 (A)	0 ± 0	141 ± 9	141 ± 6	139 ± 9	169 ± 7	307 ± 29
16 (B)		3 ± 1	5 ± 1	2 ± 1	5 ± 1	305 ± 22
13 (B)			0 ± 0	1 ± 0.5	3 ± 1	308 ± 49
125 (B)				0 ± 0	3 ± 1	334 ± 65
24a (B)					0 ± 0	318 ± 27

Divergent genes were assessed by microarray for 15 *H. pylori *isolates, three isolates from each of biopsy 15 and 16 from the mother and of biopsy 13, 125 and 24a from the three children. Isolates from the same biopsy were similar and the results are presented as the mean number of divergent genes by biopsy, averaged over comparisons between individual isolates.

^1^Standard error

The distributions of divergent genes over functional classes were nearly identical in assessments of variability of type A relative to type B isolates and of each isolate relative to the reference strains. The latter analysis did, however, yield higher estimates of variability within the functional classes, due to the overall higher frequency of divergent genes (Table 4). The DNA metabolism category had relatively high proportions of divergent genes for both analyses. This finding could be attributed to the subclass of restriction-modification genes (12 ± 1 of 76, 16%, and 33 ± 1 of 76, 43%, in the two analyses, respectively), but also to genes involved in DNA replication, recombination and repair (5 ± 0.2 of 60, 8%, and 10 ± 1 of 60, 17%, in the two analyses, respectively). The cell envelope functional class also exhibited relatively high proportions of divergent genes. These divergent genes were primarily confined to a subclass designated "other", containing outer membrane proteins (6 ± 0.3 of 59, 10%, and 14 ± 1 of 59, 24%, in the two analyses, respectively), and to genes involved in the biosynthesis of surface polysaccharides and lipopolysaccharides (4 ± 0.3 of 50, 8%, and 8 ± 1 of 50, 16%, in the two analyses, respectively). An additional class that appeared variable was cellular processes, but this variability could not be attributed to specific subclasses. The majority of the genes classified as divergent had notably not been assigned a function (82 ± 2 of 147, 56%, and 181 ± 7 of 314, 58%, in the two analyses, respectively). Genes of functional categories involved in basic metabolic functions were relatively conserved. The low number of divergent genes among the type B isolates rendered an analysis by functional class uninformative.

**Table 4:** Number of microarray divergent genes averaged by functional class and type of comparison

Functional category		Number of divergent genes for RAPD type A relative to type B isolates		Number of divergent genes for all isolates relative to the reference (26695/J99)	
Code: Description	Number of genes	Mean ± SE^1^	Percent	Mean ± SE^1^	Percent

1–7, 9–12: Basic functions^2^	635	23 ± 1	4	42 ± 5	7
8: DNA metabolism	140	17 ± 1	12	44 ± 2	31
13: Cell envelope	165	11 ± 0.3	7	24 ± 1	15
14: Cellular processes	130	9 ± 0.4	7	18 ± 2	14
15: Plasmid- and transposon-related functions	9	5 ± 0.2	56	6 ± 0.3	67
16, 17: Unknown and hypothetical	666	82 ± 2	12	181 ± 7	27
All	1,745	147 ± 4	8	314 ± 16	18

Divergent genes were assessed by microarray for 15 *H. pylori *isolates, three isolates of RAPD type A (biopsy 15) and 12 isolates of RAPD type B (biopsy 16, 13, 125 and 24a). Divergent genes by functional class were identified for the type A isolates relative to the type B isolates and for all isolates relative to the reference strains.

^1^Standard error

^2^1: Amino acid biosynthesis, 2: Purines, pyrimidines, nucleosides, and nucleotides, 3: Fatty acid and phospholipid metabolism, 4: Biosynthesis of cofactors, prosthetic groups and carriers, 5: Central intermediary metabolism, 6: Energy metabolism, 7: Transport and binding proteins, 9: Transcription, 10: Protein synthesis, 11: Protein fate, 12: Regulatory functions.

Divergent genes were dispersed throughout the chromosome, but were concentrated in the two "plasticity zones", previously named after the particular presence of strain specific genes in the two sequenced strains [11,13]. Indeed, a mean of 200 ± 7 of 314 (64%) of the oligonucleotides deemed as divergent relative to the reference strains were also classified as specific for either 26695 or J99. All studied isolates were found to contain the *cag *PAI by both microarray and PCR.

## Discussion

The present study of genome variability of *H. pylori *isolates from family members detected limited variability of clonal isolates within and between individuals. The clonal isolates have most likely been transmitted from person-to-person in the family [3-5,7]. We hypothesized that transmission and host adaptation may be associated with bacterial change by selection for fitter genetic variants. The limited variability identified for clonal isolates between the present family members does not support such change being considerable in terms of gene deletion, duplication or other substantial sequence difference.

This absence of support does, however, not disprove that transmission and host adaptation are associated with bacterial change. The modifications may be subtle, but nevertheless crucial, and not discernible by the present approach, and may act, possibly transiently, at other levels such as the transcriptome. The importance of subtle changes, for example phase-variation, is exemplified by how such modifications of lipopolysaccharide biosynthetic genes and outer membrane proteins may affect colonization efficiency in experimental models [20-22]. Furthermore, *Vibrio cholerae*, although likely to be a more infectious agent than *H. pylori*, may serve as a parallel since it has been proposed to enter a hyperinfectious state, characterized by the gene expression profile, during human infection [23].

Clonal variants were identified within individuals despite the overall low variability. *H. pylori *infection typically appears to be comprised of a predominant strain and strain variants [5,7,16,17]. The difference in genomic content among related isolates from the same individuals has been estimated to 0–2% by microarray analyses [9,16-18] and 3% or less by sequencing [8,24]. The estimates above are in accordance with the present finding of a mean of 0.1% divergent genes within individuals, considering that the microarray is not likely to contain all genes in the examined strains and that recombination between clonal isolates probably does not result in large sequence differences. A recent study reported no *H. pylori *genomic divergence in human volunteers that had been experimentally infected [16]. The authors proposed a study like the present to investigate the diversity of *H. pylori *populations during natural transmission. Apparently, also our study is limited in this aspect due to the sparse genetic diversity detected.

The mother was found to harbor two clearly distinguishable strains, one of which was also isolated from the children. The finding of a strain in the mother not being detected in the children could be explained by the strain being less infectious [20,21,25], by chance or by the strain remaining unsampled in the children. Co-infections with distinct strains have been hypothesized to be more common in high- than in low-prevalence regions [26] and can facilitate genetic diversification by recombination [6,16,18,24]. The *mutY *sequences showed signs of a past recombination event between type A and type B strains, showing that these strains have encountered each other in at least one family member.

The genomic variability was most pronounced when measured relative to the reference strains, with a mean estimate of 18% divergent genes. This estimate is in accordance with previous microarray data of unrelated isolates from different individuals, where 12–18% of the genes were reported to be dispensable in each isolate [13] and 22–32% were deemed as absent in at least one isolate [13-16]. In three sequenced *H. pylori *strains, 3–7% of the genes were specific to each strain [9,11]. The higher estimates above could be explained by strain-specific genes from two strains being included in the denominator, the same gene varying in sequence or a closer evolutionary relation between the sequenced strains.

The variability between different strains was associated with gene functional classes related to DNA metabolism and the cell envelope. The diversity of genes involved in DNA metabolism may reflect the importance of these systems in regulating the genetic diversity of *H. pylori *[14,27,28]. Molecules on the bacterial surface are exposed to the host and may thus need to undergo antigenic variation in contrast to gene products involved in basic metabolism. This notion could explain the diversity found for genes encoding outer membrane proteins [14,21,22,28] and for genes involved in biosynthesis of surface polysaccharides and lipopolysaccharides [14,20,28,29]. Moreover, the cellular processes functional class appeared variable as described before [14,28]. Previously described plasticity zones in the sequenced strains were discernible in the present data [11,13,14].

The limitations of the present study should be considered when interpreting the findings. First, detailed studies of human *H. pylori *isolates are, like ours, commonly restrained by a relatively sparse number of samples per person and time point with largely unknown representativeness. There are, however, no indications that the examined isolates would exhibit atypical properties. Second, microarray hybridization will not detect variability in sequences not represented by the array, minor mutations or altered arrangements of genes on the chromosome. Furthermore, some genes may have been misclassified as divergent or non-divergent. However, the evaluations of the microarray results point to that misclassification is unlikely to have influenced our conclusions significantly. The present conclusions are further based on patterns of variability over averaged comparisons between all isolates and are hence less prone to misinterpretation due to technical artifacts in the hybridizations of individual isolates. Previous comparable studies have described approximately the same magnitude of variability [13-18], concentrated to the same functional classes as observed in the present study [11,13-16,28]. This consistency corroborates the properness of the present data and thus also of this novel investigation of genome variability of clonal *H. pylori *isolates from family members by using microarray.

## Conclusion

The present study does not support that transmission and host adaptation are associated with substantial sequence diversity in the *H. pylori *genome. However, important phenotypic modifications may be determined by undetected subtler genomic changes, such as phase-variation. The considerable variability between different strains is associated with gene functional classes related to DNA metabolism and the cell envelope. These findings can aid further exploration of *H. pylori *genetic diversity and adaptation.

## Methods

### Subjects and samples

Family members were invited to participate in an epidemiological study and contributed questionnaire information and blood for serological *H. pylori *infection status determination [4]. As a follow-up, seropositive individuals with gastrointestinal complaints were offered gastroscopy and treatment with informed consent. The typical symptom was reported abdominal pain, but symptoms were not monitored systematically. Approval was obtained from the ethics committee at Karolinska Institutet (Reference numbers 37/97, 252/98, 99/119). *H. pylori *was cultured from biopsies and typed with PCR-based methods [5]. For the present study, a mother and her three children originating from South America were selected for more detailed genetic analysis of their clonal *H. pylori *isolates (Table 1). There was no father present in the family. The inclusion of several children of different ages was regarded to enable study of a number of transmission events after time periods of different lengths. *H. pylori *isolates were obtained from single colonies picked at random from standard primary plate cultures of five gastric biopsies. Bacterial genomic DNA was prepared from 11–13 isolates per biopsy (n = 59) (QIAamp DNA Mini; Qiagen, Hilden, Germany). Three random isolates per biopsy, yielding in total 15 isolates, were analyzed in more detail by sequencing and microarray.

### PCR and sequencing

All PCR applications were performed under standard conditions unless stated otherwise (DyNAzyme DNA polymerase; Finnzyme, Espoo, Finland). The relatedness of all 59 isolates was assessed by random amplified polymorphic DNA (RAPD), using the primers 1283 and 1290 as described previously [30]. The presence or absence of the *cag *pathogenicity island (PAI) was determined by amplification in either of two reactions with primer pairs within the *cagA *gene or flanking the *cag *PAI [31]. For each of the 15 isolates selected for more detailed examination, approximately 2,500 base pairs were sequenced from five PCR fragments within the genes *hsdS5 *(HP0790), *cag2 *(HP0521), *flaB *(HP0115),*mutY *(HP0142) and *ureI *(HP0071) (Additional file 1). The loci were selected based on their inclusion in previous studies of *H. pylori *sequence diversity [8,10,19]. The quality of the PCR products was verified by agarose gel electrophoresis and both strands were sequenced (BigDye Terminator 3.1 and ABI 3100 Instrument; Applied Biosystems, Foster City, CA, USA) prior to analysis (Vector NTI Advance 9.0; Invitrogen/InforMax, Carlsbad, CA, USA).

### Microarray

The *H. pylori *microarray consisted of 1,920 50 mer oligonucleotides (MWG Biotech, Edensberg, Germany) spotted in triplicates on glass slides (Qarray arrayer; Genetix, Boston, MA, USA; MWG Epoxy Slides; MWG Biotech, Edensberg, Germany). The oligonucleotides represented the genes of the fully sequenced strains 26695 and J99 [11,32]. Gene functional categories were assigned according to a previously revised annotation [28] and exhaustive information for individual genes is available from the corresponding database [33]. The oligonucleotide sequences were evaluated by BLAST analysis against the 26695 and J99 genomes. Seventy-three oligonucleotides were excluded because of sequence similarity to more than one locus in either genome and 359 were specific to either 26695 or J99 (E-value ≤ 0.0004 or sequence similarity ≥ 50%). Furthermore, the final analysis excluded 43 *Arabidopsis thaliana *negative controls and 59 oligonucleotides that in the analysis did not yield any data for at least one isolate after filtering of poor quality spots. Thus, the final statistical analysis considered 1,745 oligonucleotides.

The microarray system was evaluated by test hybridizations using DNA from 26695 and J99. First, in two self-self hybridizations with a mixture of 26695 and J99, the specificity was estimated to 100% (1,745 of 1,745) and there were no signs of gene- or intensity-dependent bias of the intensity ratios. Second, four hybridizations including two dye-swaps where 26695 and J99 were differentially labeled and hybridized against each other yielded a sensitivity of 81% (292 of 359). Furthermore, the microarray results for one of the present test isolates relative to the reference sample were confirmed by PCR and sequencing. Genes classified as divergent were randomly selected, covering the whole range of intensity ratios below 0.5. Amplification was frequently unsuccessful (13 of 30, 43%) compared to positive controls, corroborating the absence of these genes, while the sequence of the rest differed by 20% on average.

For each of the 15 test isolates, four hybridizations including two dye-swaps were performed, hence yielding a total of 60 microarray hybridizations and up to 12 measurements per oligonucleotide and isolate. The microarray procedure was developed from a previously published protocol [13]. Briefly, a Klenow enzyme reaction primed with random octamers (BioPrime DNA Labeling System; Invitrogen/Life Technologies, Carlsbad, CA, USA) incorporated aminoallyl dUTP (Sigma Aldrich, St. Louis, MO, USA) using 2 μg genomic DNA templates of the test isolate and of the reference sample, the latter consisting of an equimolar mixture of 26695 and J99. Non-incorporated nucleotides were removed (Microcon 30; Millipore, Billerica, MA, USA), the DNA was covalently linked to monofunctional NHS-ester Cy3 or Cy5 (GE Healthcare/Amersham Biosciences, Uppsala, Sweden) and the samples were purified (QIAquick PCR Purification Kit; Qiagen). An automated hybridization station (TECAN HS400; Tecan Group Ltd., Maennedorf, Switzerland) managed the hybridization (MWG Hybridization Buffer; MWG Biotech; 42°C, 16 h) and washing (5 min of each: 1× SSC, 0.2% SDS; 0.1× SSC, 0.2% SDS; twice 0.1× SSC). The microarrays were scanned at 532 nm and 635 nm (GenePix 4000B scanner; Molecular Devices/Axon Instruments, Union City, CA, USA).

### Microarray analysis

The hybridization results were subjected to an initial quality control and the intensities were quantified (GenePix Pro 5.0; Molecular Devices/Axon Instruments). Spots of poor quality or low intensity (70% of pixels below two standard deviations of the background in both channels) were excluded. The median background intensity was subtracted from the median foreground intensity and the intensity ratio of the test isolate relative to the reference sample was calculated (GeneSpring 7.0; Agilent Technologies/SiliconGenetics, Palo Alto, CA, USA). Ratios were normalized to the median intensity of 1,005 spots with non-normalized ratios between 0.5 and 2 in all 66 hybridizations, corresponding to the density of data points around a ratio of one. The normalization procedure of choice accommodated the skewness of the data towards ratios below one, originating in the sequence differences of the test isolate relative to the reference strains, and the relatively large number of spots used for the normalization benefited its stability. The normalization was carried out separately for each print-tip to take differences in spotting performance into account and repeated measurements were averaged.

To identify divergent genes in each of the 105 individual combinations of the test isolates, t-tests were performed without assuming equal variances [34]. The Benjamini and Hochberg false discovery rate corrected for multiple testing. With an error rate of 0.05, this approach is expected to control the rate of false discoveries to 5% of divergent genes. Additionally, genes had to exhibit at least a two-fold difference in intensity to be classified as divergent. For assessment of variability relative to the reference strains 26695 and J99, t-tests were applied with the same settings as above for multiple testing correction and two-fold change [34]. Means and standard errors (± SE) of the numbers of divergent genes were calculated as summary measures.

## Authors' contributions

MK designed and coordinated the study, carried out the experiments and wrote the manuscript. SR participated in the sequencing and microarray experiments and the design and analysis thereof. IK participated in the design and analysis of the microarray experiments. AL participated in the sequence experiments and the design thereof. YT participated in the collection of biological samples and the design of the study. MG participated in the collection of biological samples and the design of the study. LE conceived the study and participated in its design and coordination. All authors have read and approved the final manuscript.

## Supplementary Material

Additional File 1 Primer information and sequences Click here for file

Additional File 2 Microarray data for all isolates in the present study and lists of identified divergent genes Click here for file
